# Hybrid QM/MM study of FMO complex with polarized protein-specific charge

**DOI:** 10.1038/srep17096

**Published:** 2015-11-27

**Authors:** Xiangyu Jia, Ye Mei, John Z.H. Zhang, Yan Mo

**Affiliations:** 1 State Key Laboratory of Precision Spectroscopy and Department of Physics and Institute of Theoretical and Computational Science, East China Normal University, Shanghai 200062, China; 2 NYU-ECNU Center for Computational Chemistry at NYU Shanghai, Shanghai 200062, China; 3 Department of Chemistry, New York University, New York, NY 10003

## Abstract

The Fenna-Matthews-Olson (FMO) light-harvesting complex is now one of the primary model systems for the study of excitation energy transfer (EET). However, the mechanism of the EET in this system is still controversial. In this work, molecular dynamics simulations and the electrostatic-embedding quantum-mechanics/molecular-mechanics single-point calculations have been employed to predict the energy transfer pathways utilizing the polarized protein-specific charge (PPC), which provides a more realistic description of Coulomb interaction potential in the protein than conventional mean-field charge scheme. The recently discovered eighth pigment has also been included in this study. Comparing with the conventional mean-field charges, more stable structures of FMO complex were found under PPC scheme during molecular dynamic simulation. Based on the electronic structure calculations, an exciton model was constructed to consider the couplings during excitation. The results show that pigments 3 and 4 dominate the lowest exciton levels whereas the highest exciton level are mainly constituted of pigments 1 and 6. This observation agrees well with the assumption based on the spatial distribution of the pigments. Moreover, the obtained spectral density in this study gives a reliable description of the diverse local environment embedding each pigment.

In photosynthesis, sunlight is captured by light-harvesting antenna system, and then the electronic excitation energy is transferred from the antenna to the reaction center (RC) and converted into a more stable chemical form. Fenna-Matthews-Olson (FMO) complex is one of the extensively studied model systems since it was the first pigment-protein complex of which the X-ray structure was obtained decades ago^1^. Recently, the X-ray structure of FMO from *Chlorobaculum tepidum* has been revised. Meanwhile, the high resolution (1.3 Å) X-ray data of FMO from *Prosthecochloris aestuarii* has been collected^2^. Besides the discovery of the eighth pigment, those data provided more structural detail for the subsequent theoretical investigation. The FMO complex is a trimer consisting of three identical subunits (monomers) in *C*_3_ symmetry. In each subunit, seven BChls are enveloped by their corresponding protein chain, while the eighth BChl resides outside the envelop and is situated in a cleft in the protein surface (see Fig. 1). It has been suggested that the specific local environment is responsible for the arrangement of pigments spatially and energetically, leading to a funnel-like architecture to mediate the energy transfer efficiently^3^,^4^,^5^,^6^,^7^,^8^,^9^,^10^,^11^,^12^,^13^,^14^,^15^,^16^. Several studies have been dedicated to the individual effect of local environments and showed that the dynamic protein environment, especially the organization of the charged residues, is the major modulator of site energy shift^17^,^18^,^19^. Early studies on site energy ladder concerned about only seven BChls, as summarized in ref. 20. In the absence of BChl 8, BChls 1 and 6 are assumed to have the highest site energies because they are believed to be the nearest pigments to the antenna which captures the sunlight^21^,^22^,^23^, while BChl 3, which is the closest one to the RC, is supposed to have the lowest site energy^22^,^24^,^25^. Along with the discovery of the eighth BChl, the role of this pigment in the excitation energy transfer has been investigated^8^,^9^,^10^,^11^,^12^,^26^,^27^. These studies suggested that BChl 8, which resides in the proximity of the chlorosome, has a very large transition energy. It thus receives a significant part of excitation from chlorosome and plays an important role in energy transfer process. Note that these sets of site energies are quite different from each other due that the distinct methods were employed in these studies, leading to diverse pictures of transport dynamics. This inconsistency may be due to the inaccuracy of force field parameters, neglect of the dynamic nature of protein environments, insufficient sampling of protein conformations, and semiempirical methods used.

In this work, we constructed an exciton model for accurate description of the optical properties of light-harvesting system and energy transfer dynamics. Molecular dynamics (MD) simulation has been combined with quantum mechanics/molecular mechanics (QM/MM) calculation to study the time evolution of site energies. The polarized protein-specific charge (PPC), rather than the conventional mean-field charge from pairwise force field, was utilized in both the MD simulation and the site energy calculations. The result depicts a more reasonable energy ladder, which agrees with the experimental observation and phenomenological description based on the seven-site model^6^. It can be conjectured from this study that pigment 8 may not play an important role in the excitation energy transfer (EET) through FMO complex^9^,^12^. Furthermore, the spectral density is simulated. The resulting 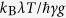, a key parameter for energy transfer efficiency, is in the optimal range, enabling a nearly perfect energy transfer^28^.

## Results

### The structure of the complex is stable during the simulation

**Figure 2:**
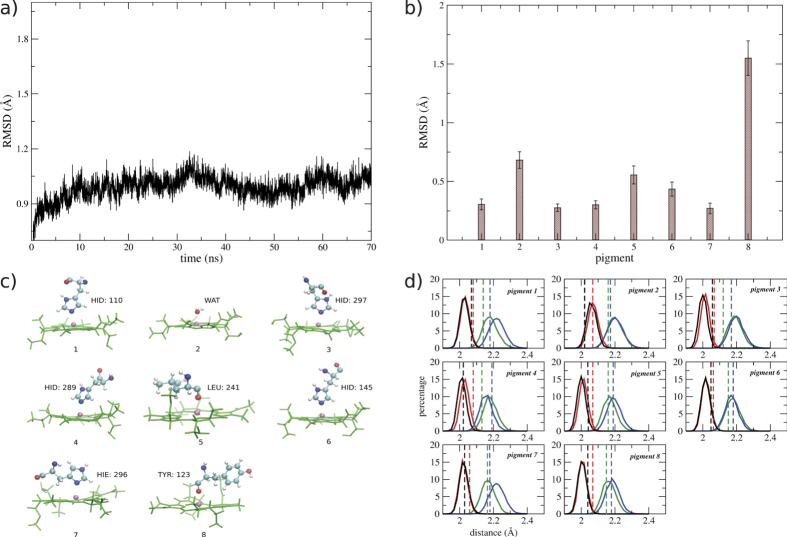
(**a**) Variations of the RMSD of protein’s backbone heavy atoms for the FMO complex during the MD simulation. (**b**) The RMSD of each pigment from its corresponding crystal structure. (**c**) The local environment of each pigment. (**d**) Distributions of distances between the magnesium and its corresponding four nitrogen atoms in each pigment. The dash lines represent distances in the crystal structure.

The structural analysis from several perspectives shows that the FMO complex remained stable during the MD simulation. Shown in Fig. 2a is the root mean square deviations (RMSD) of the protein backbone heavy atoms from the crystal structure. It reaches the equilibrium in 10 ns and fluctuates around 1.0 Å whereafter. The RMSD of the protein in a 70 ns long MD simulation utilizing AMBER03 mean-field charges was also calculated (see Supplementary Fig. S1). By comparison, the protein is found more stable under PPC than it is under AMBER charges. The flexible pythyl tail was excluded in the RMSD analysis of individual pigment. As shown in Fig. 2b, the RMSD of BChls 1, 3, 4, 6 and 7 are around 0.5 Å, while those of BChls 2 and 5 fluctuate between 0.5 Å and 1.0 Å. This difference among BChls is induced by their specific local environment, which is depicted in Fig. 2c. There is a water molecule around BChl 2. BChl 5 interacts with the main chain oxygen atom of LEU. BChls 1, 3, 4, 6 and 7 has strong interaction with each side chain HIS residue, respectively. Therefore, BChls 1, 3, 4, 6 and 7 are more stable than BChls 2 and 5. Because BChl 8 is located at the interface between two monomers, its property and function might be significantly influenced by the solvent^18^, thus its RMSD suffers a large fluctuation around 1.5 Å. Distributions of the distance between Mg and nitrogen atoms for each pigment are shown in Fig. 2d. The corresponding distances in the crystal structure are plotted as the vertical dashed lines. This figure clearly shows that all the distances exhibit a Gaussian-like distribution. The full width at half maximum (FWHM) of these distributions are about 0.1–0.15 Å and the peak positions deviate from their corresponding crystal structure values by less than 0.05 Å. It suggests that the magnesium ions were tightly bonded to their surrounding nitrogen atoms during the whole simulation. This stable trajectory under PPC thus provides a more reliable structural ensemble, which is critical to the calculations of site energies and coupling between the pigments.

### The determination of energy transfer pathways

It has been shown that the dynamics of excitation energy heavily depends on the arrangement of the pigments in the energy ladder and also the excitonic couplings between pigments. The calculated average site energies of individual pigment are tabulated in Table 1 (diagonal terms), from which an evident and reasonable energy ladder is implied: the lowest site energy is found at BChl 3, as reported in most studies based on either a seven-site model or an eight-site model^20^; BChls 1 and 6 have the highest site energies. Therefore, BChls 1 and 6 are the most probable sites for excitations. Although the BChl 8 is more close to the chlorosome, its site energy is found in this study to be lower than BChls 1 and 6. The site energies based on the mean-field AMBER charges were also calculated and compared with those obtained by PPC (see Supplementary Fig. S2). A wider distribution of site energies is found under PPC, which is consistent with that obtained by fitting the experimental data^3^.

The intra-monomer excitonic couplings were calculated by Eq. 1 (see Methods) and averaged along the MD trajectory. The averaged coupling strengths were also listed in Table 1 (off–diagonal terms). Since BChl 8 is more close to BChl 1 of the nearest-neighboring monomer, the inter-monomer excitonic couplings between BChl 8 and the seven BChls of the neighboring monomer are also listed in Table 1 (denoted as 8′ for differentiation). In principle, the strongest couplings are to BChls with neighboring indexes except for coupling of 4–7, which is more than twice of that between BChl 6 and 7. For BChl 8, all intra-monomer couplings are very weak, while the inter-monomer coupling between BChl 8′ and BChl 1 is relatively strong.

**Figure 3:**
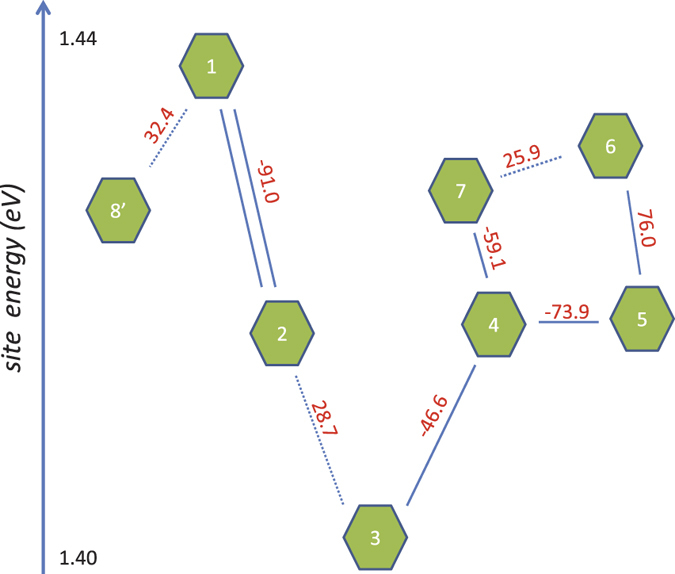
The diagram of energy ladder based on the calculated site energies and the coupling strength. The strength of electronic coupling among pigments are ranked orderly by double solid line, solid line and dash line.

Combining the excitonic couplings with the site energies for FMO trimer, a full description of the energy ladder is given in Fig. 3. Two independent pathways of EET are proposed. One way is from BChl 1 to 2, and the other way is from BChl 6 to BChls 7, 5, and 4, then the excitation energy is finally trapped by BChl 3 and delivered to the RC. This is in agreement with the previous experimental observations and phenomenological descriptions based on the experimental results^29^,^30^.

### The role of the eighth pigment

**Figure 4:**
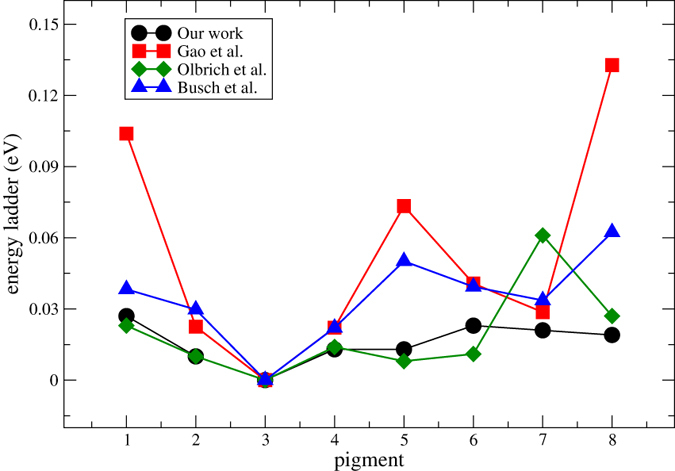
Comparison of site energies obtained in this work (black circles) with previous studies by Busch *et al*.^8^ (blue triangles), Olbrich *et al*.^9^ (green diamond), and Gao *et al*.^10^ (red squares).

The calculated site energies are depicted in Fig. 4, as well as three recent studies in which the eighth BChl was also taken into account^8^,^9^,^10^. It is worth noted that what we consider here is the relative values among pigments. BChl 3 possesses the lowest site energy in all the studies, thus its site energy were set to zero for convenient comparison. Olbrich *et al.*^9^ showed a similar trend as our work except that the site energy of BChl 7 in their work is much larger and that of BChl 8 is higher than BChls 1 and 6. Busch *et al.*^8^ and Zhao *et al.*^10^ showed another trend with a larger energy gap and the highest site energy was found on BChl 8. They suggested that BChl 8 might act like a linker to accept excitation energy from chlorosome and transfer it to the remainder pigment-protein complex. This might cause a dramatic change in EET pathway selection and EET efficiency. Theoretical studies that adopted the aforementioned site energy values in a calculation of population transfer found that the initial excitation on BChl 8 might lead to a suppression of one of the EET pathways, which involves BChls 4, 5, 6 and 7^11^. Furthermore, the initial excitation on BChl 8 also results in a suppression of population oscillations and a relatively low EET efficiency^12^. The suppression of population oscillations is due to the weak coupling of BChl 8 to other BChls, either intra- or inter-monomer, and is in contrast to the corresponding experimental observations.

In our study, the site energy of BChl 8 is lower than those of BChls 1 and 6. By diagonalizing the electronic Hamiltonian (listed in Table 1), the delocalized exciton states are attained. The eigenvalues and each pigment’s contributions to exciton levels are tabulated in Table 2. Adopting similar strategy as in a previous study^31^, the data in Table 2 were used to calculate the simulated absorption spectrum, which is in good agreement with the experimental one (see Supplementary Fig. S3). The delocalized excitations can also be analyzed. We can see that BChls 3 and 4 contribute most to the lowest exciton level. The highest exciton level is mainly comprise of contributions from excitation states of BChls 1 and 6. The contribution from excitation states of BChl 8 is trivial. We then conclude that although the eighth pigment is the nearest pigment to the chlorosome, it does not play an important role in excitation energy transfer through FMO complex, which is different from several previous studies^8^,^9^,^10^. To understand the actual biofunctional role of the eighth pigment, we need to include part of the chlorosome in atomic detail during theoretical investigation^26^.

### The energy transfer efficiency is in optimal regime

**Figure 5:**
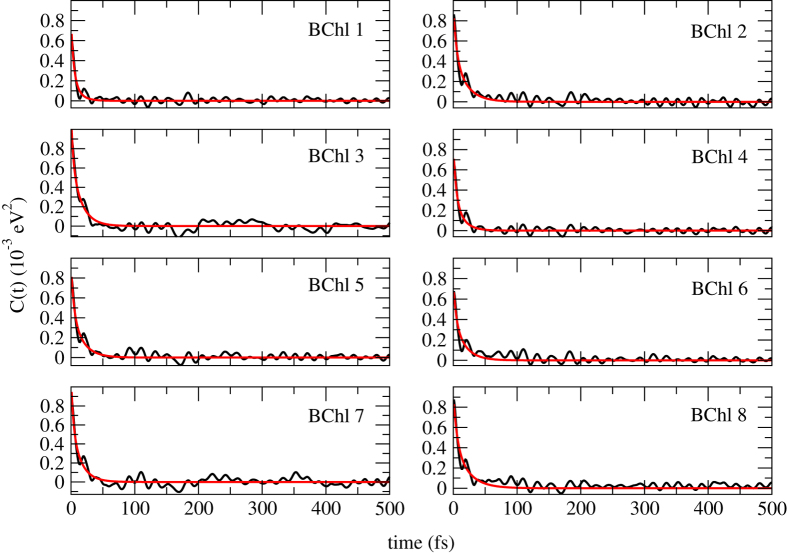
The autocorrelation function for individual BChls obtained from the MD simulations (black) and the fitted one using a biexponential function (red).

**Figure 6:**
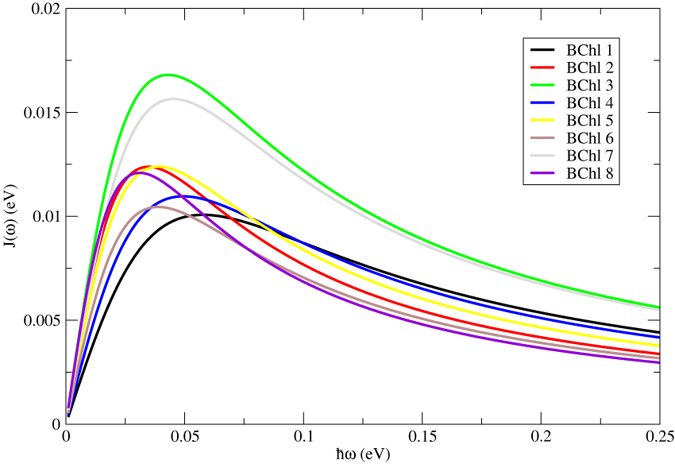
The simulated spectral densities using the Drude model for individual BChls in the FMO complex.

The autocorrelation function of site energies of each pigment was obtained based on a 20-ps production run with a time step of 0.5 fs in order to address the fast modulation. To validate the achievement of convergence, averages of the site energies and their corresponding standard deviations from this 20-ps production run (40,000 snapshots with a time interval of 0.5 fs) and a 200-ps production run (also 40,000 snapshots in total but with a time interval of 5 fs) are shown in the Supplementary Fig. S4. As can be seen in this figure, both the site energies and their fluctuations from those two trajectories show high similarity, indicating that the convergence has been reached in 20 ps. The autocorrelation functions of the site energy for each BChls and the corresponding fitted curve for FMO complex are shown in Fig. 5. The autocorrelation functions (black lines) decay to zero gradually and converge very well. For simplicity, in this work, the Drude model (see Eq. 5) was utilized to simulate the spectral density. The calculated autocorrelation function are fitted to a biexponential function (see Eq. 6), and the fitted parameters are listed in Table 3. According to Tian *et al.*^32^, 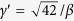, and 1/*γ* ′ is supposed to be around 4 fs at 300 K, since it only depends on temperature. In the current work, the averaged timescale of the fast modulation is 4.20 fs, which is very close to the theoretically estimated value. The timescales of the slow modulation and the reorganization energies vary among pigments because each pigment is interacting with its specific local environment^18^. The spectral densities for individual pigment are shown in Fig. 6. The peak positions of BChls 1, 3, 4, 6 and 7 are similar, since they are well coordinated to the histidine residues, except that the amplitudes of BChls 3 and 7 are much larger. The spectral density of BChls 2, 5 and 8 share another trend, because the interactions between these pigments and their environments are weak. In addition, the averaged spectrum density, as well as those from previous works^3^,^33^, is shown in the Supplementary Fig. S5.

Taking an average over eight BChls gives a total reorganization energy *λ* = 102 cm^−1^ and a cutoff frequency *γ* = 330 cm^−1^ at *T* = 300 *K*. The extensively used values are the experimentally estimated ones at *T* = 298 *K*, *λ* = 35 cm^−1^ and *γ* = 50–166 cm^−1^, which were extracted from low-temperature absorption spectrum by using an Ohmic spectrum density model^34^. To study the optimality and robustness of quantum transport in photosynthetic complexes, Mohseni *et al.* proposed a single parameter 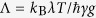 (g is the average excitonic energy gap) for determining the energy transfer efficiency. In our study, Λ = 1.05, indicating an optimal energy transfer efficiency.

## Discussion

To investigate the dynamic effect of the environment on the excitation energy landscape of FMO complex, with special focus on the role of the eighth pigment, we have constructed an excitation model based on MD simulations and QM/MM methods. In this model, PPC scheme is used for more realistic description of the protein interactions. Taking the advantage of PPC scheme, the following structure analysis showed that the whole protein–pigment complex remained stable during the MD simulation, and the electronic calculations based on the structural ensemble gave a more reasonable energy ladder. From the detailed analysis of the site energy and the excitonic coupling, we can draw a conclusion that the role of the eighth pigment is not as important as has been suggested in previous studies. More experimental and theoretical studies are desired to unveil its specific role in the light harvesting process.

We noticed that the site energy values we obtained are lower than those from previous studies^8^,^9^,^10^. The main cause is the different combinations of force fields and quantum chemistry approaches employed. In ref. 23, List *et al.* compared the energy ladders calculated using ZINDO, CIS and TD–DFT (with several functionals). They found that ZINDO shows a reasonable trend across the pigments similar to CAM–B3LYP, but the site energies are significantly underestimated. In a recent study, Chandrasekaran *et al.*^35^ also compared the site energies and spectral densities calculated with different combination of CHARMM or AMBER force field and the ZINDO/S or TDDFT quantum chemistry methods. They concluded that CHARMM force field always shows a wider spread in energy ladders, resulting in a larger value of spectral density at low frequencies. Moreover, in our study, the protein polarization effect was explicitly taken into account by employing the polarized protein specific charge instead of the mean-field charge schemes as has been used in other studies. We also noticed that the reorganization energy and cutoff frequency deviate from the frequently-used theoretical values, since those values were extracted from available experimental measured spectra^34^. The spectral density model used has been proved to be insufficient for describing the high EET efficiency recently^28^. In our study, we have used a simple and more accurate bath spectral model and the effective parameter obtained indicated that the system is in an optimal environment–assisted quantum transport regime.

## Methods

### System preparation

The FMO trimer structure was built based on the crystal structure 3ENI downloaded from the Protein Data Bank. The ions and crystallographic water molecules were reserved. All the Asp and Glu residues were deprotonated, while the Lys and Arg residues were protonated. For those His residues that coordinate to BChls, the protonation states were manually assigned: His 110, 145, 289 and 297 were protonated at the *N*_*ε*_ while His 296 was assumed to be protonated at the *δ* position. Hydrogen atoms were added using the LEaP module in AmberTools package. The pigment structure was optimized at B3LYP/6-31G* level. The RESP charge^36^,^37^ fitted to the electronic structure calculated at the same QM level and the general AMBER force field^38^ were assigned to the pigment. AMBER03^39^ force field was applied to the protein. The FMO complex was soaked in a periodic truncated octahedral box of TIP3P water with the minimal distance between the protein and the boundary of the cell box no less than 15 Å. The whole system was neutralized with counter-ions.

### PPC

Reliability of this study depends on the proper use of the force field, extent of sampling in the phase space and accurate description of the effect of protein environment. In most, if not all, of the previous studies, the protein is assigned mean-field charges from pairwise force field. In those charge schemes, the polarization effect is taken into account implicitly and the atomic charges are residue-specific and invariant to the alternation of the chemical environment. Employing these charges in the QM/MM calculation of the excitation energy might underestimates the QM/MM coupling^40^,^41^,^42^,^43^,^44^. We applied here a new charge scheme proposed recently, termed polarized protein-specific charge^40^,^45^. In this scheme, the electrostatic potential of the whole protein is calculated at QM level by molecular fractionation with conjugate caps method^46^,^47^. Then PPC is obtained by restrained fitting to the electrostatic potential (RESP)^36^,^37^. This fitting method is consistent with that in the development of AMBER force field and believed to be compatible with other AMBER parameters. The advantage of PPC over mean-field charges has been proven in several studies and has been reviewed recently in ref. 48. AMBER charge has also been utilized in this work. However, the trajectory was less stable than that from PPC. Therefore, we mainly focused on the trajectory of PPC. Some comparisons between PPC and AMBER03 mean-field charges can be found in the Supplementary Information.

Starting with the AMBER charge, the induced charges on the solute–solvent interface were calculated by solving the Poisson–Boltzmann equation utilizing Delphi program^49^. The internal dielectric constant was set to unity and that of the solvent to 80. Grid density was set to 3.5 grids/Å. The protein was cut at the peptide bond position and a pair of conjugated caps was added to saturate covalent bonds and also mimic the immediate chemical environment. QM calculation was performed for each fragment in the electric field generated by other fragments and induced charges on the solute-solvent interface. RESP program in AmberTools suite package with some in-house modifications was applied^45^. The new charges were used in the next cycle of Poisson-Boltzmann calculation. Atomic and induced charges were calculated iteratively until convergence had been reached, which was determined by the variation of dipole moment of the protein and the root-mean-square deviation of surface charges. Solvation and many-body effects had been naturally included via iteration. All the QM calculations in the PPC fitting were carried out at B3LYP/6-31G* level utilizing Gaussian 09 package^50^.

### MD simulations

In the initial minimization stage, the protein and pigments were restrained and other parts were optimized by the steep descent method followed by the conjugated gradient method. Then, the whole system was relaxed without any restraint applied. The thoroughly relaxed structures were used for PPC fitting. A six-stage heating-up in 1.2 ns altogether was applied to avoid blowup of the system. The time step was 1 fs during heating. A 2-ns simulation in canonical ensemble was carried out to equilibrate the whole system to the target temperature followed by a 70-ns simulation in NPT ensemble with a 2-fs time step. SHAKE algorithm was applied to constrain all the covalent bonds involving hydrogen atoms. The temperature was regulated by Langevin dynamics^51^ with a collision frequency of 1.0 ps^−1^. The pressure was regulated using Berendsen’s barostat^52^. Particle mesh Ewald^53^ with a cutoff of 12.0 Å in real space was utilized to calculate long range Coulomb interaction. The van der Waals interaction was truncated at 12.0 Å. Finally, a 200 ps production run with a time step of 1 fs was carried out. Trajectory was collected every 5 steps and the saved conformations were used for the subsequent calculations of site energies and electronic couplings. To address the fast modulation (~4 fs), an extra 20 ps production run with a time step of 0.5 fs was carried out. Totally 40,000 snapshots were collected for the spectral density simulation. The molecular dynamics simulation was carried out by AMBER 11 package^54^.

### Calculation of site energies and electronic couplings

The site energies were calculated using semi-empirical ZINDO/S-CIS method, as implemented in ORCA program (version 2.9.1)^55^. Ten highest occupied and ten lowest unoccupied states were chosen for the configuration interaction. Before the electronic structure calculations, the whole system was imaged back to the central unit cell with the pigments located at the center of the simulation box one-by-one. The pythyl tail was replaced by a hydrogen atom for computational efficiency. The surrounding environment (the remaining pigments, proteins, water molecules and ions etc) was treated as atomic charges. The ZINDO/S-CIS method has been extensively used in the electronic-structure calculations of light-harvesting systems^9^,^56^, for its outstanding balance between accuracy and efficiency.

The coupling between the pigments can be described with the point dipole approximation^10^,^17^:


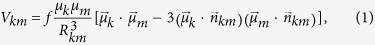


where *f* is the protein screening factor, *μ*_*k*_ and *μ*_*m*_ are the strengths of transition dipoles for the *k*th and *m*th pigment and 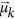 and 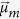 are their corresponding unit vectors, *R*_*ij*_ is the distance between transition dipoles with the direction 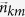. The direction of transition dipole was taken from ND to NB according to the nomenclature of PDB. *R*_*km*_ was taken as the distance between the magnesium atoms in the *k*th and *m*th pigments. Some approximations were adopted: *μ*_*k*_*μ*_*m*_ = 37.5 *D*^2^ and *f* = 0.8 as in some previous studies^10^,^17^.

### Evaluation of correlation functions and spectral densities

In order to calculate the spectral density, the autocorrelation function for each pigment was calculated by the following equation:


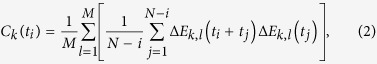


where Δ*E*_*k*,*l*_(*t*_*i*_) is the energy difference at time steps *t*_*i*_ for BChl *k* in monomer *l*, which is defined as


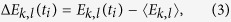


where *E*_*k*,*l*_(*t*_*i*_) and 〈*E*_*k*,*l*_〉 are the instantaneous and mean value of site energy, respectively.

The autocorrelation function *C*(*t*) can be related to the spectral density *J*(*ω*) via the fluctuation-dissipation theorem:


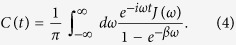


Here *β* is the inverse temperature denoted by *β* = 1/(*k*_B_*T*). In this work, we adopt the Drude model for the spectral density,


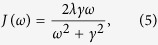


where *γ* is the cut-off frequency and *λ* is the reorganization energy which corresponds to the system-bath coupling strength. The correlation function can be approximated by the best biexponential function with the Markovian white noise residue (WNR) ansatz, as reported by Tian *et al.*^32^, i.e.,


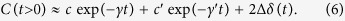


The involved parameters can be obtained by exploiting the Laurent expansion and [1/1] padé approximation


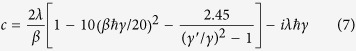


and


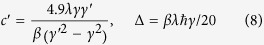


In this work, Eq. 6 combined with Eqs 7 and 8 was used for fitting the correlation function. Substituting *γ*, *γ*′ and *λ* into Eq. 5, the spectral density can be calculated.

## Additional Information

**How to cite this article**: Jia, X. *et al.* Hybrid QM/MM study of FMO complex with polarized protein-specific charge. *Sci. Rep.* **5**, 17096; doi: 10.1038/srep17096 (2015).

## Supplementary Material

Supplementary Information

## Figures and Tables

**Figure 1:**
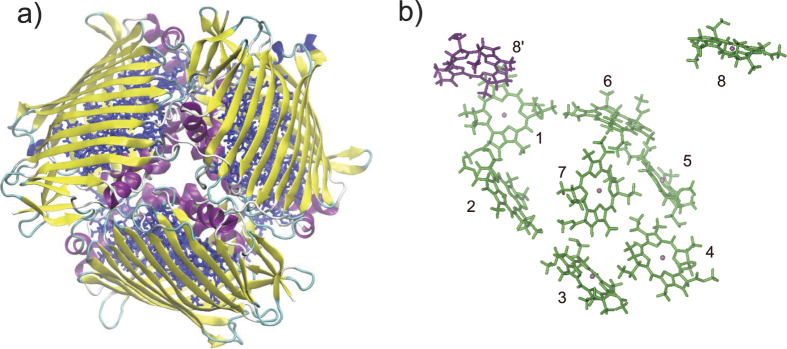
(**a**) The FMO trimer with protein backbone. All BChls are in blue. (**b**) The spatial arrangement of BChls. The eight BChls of the same monomer are colored in green where the BChl 8′ of the nearest–neighboring monomer is colored in purple.

**Table 1:** Calculated site energies (diagonal) and electronic coupling (off–diagonal) of FMO trimer in units of cm^−1^.

Pigment	1	2	3	4	5	6	7	8	8′
1	11550	−91.0	4.1	−6.3	6.3	−8.8	−7.8	0.2	32.4
2	−91.0	11413	28.7	8.2	1.0	8.8	3.4	0.9	6.3
3	4.1	28.7	11332	−46.6	−4.4	−9.3	1.3	2.2	1.3
4	−6.3	8.2	−46.6	11437	−73.9	−17.7	−59.1	−2.0	−1.9
5	6.3	1.0	−4.4	−73.9	11437	76.0	−3.1	6.1	4.2
6	−8.8	8.8	−9.3	−17.7	76.0	11518	25.9	−4.2	−11.6
7	−7.8	3.4	1.3	−59.1	−3.1	25.9	11501	−7.8	−11.9
8	0.2	0.9	2.2	−2.0	6.1	−4.2	−7.8	—	—
8′	32.4	6.3	1.3	−1.9	4.2	−11.6	−11.9	—	11486

**Table 2:** Exciton energies (in units of eV) and the contribution of each pigment to exciton levels.

exciton level	exciton energies	BChl 1	BChl 2	BChl 3	BChl 4	BChl 5	BChl 6	BChl 7	BChl 8
1	1.439	0.38	0.09	0.00	0.06	0.08	0.23	0.10	0.04
2	1.438	0.38	0.08	0.00	0.14	0.15	0.18	0.06	0.01
3	1.427	0.00	0.00	0.01	0.08	0.07	0.25	0.57	0.02
4	1.424	0.02	0.07	0.02	0.05	0.02	0.03	0.00	0.80
5	1.419	0.00	0.00	0.05	0.29	0.17	0.20	0.19	0.10
6	1.411	0.16	0.49	0.23	0.00	0.07	0.02	0.01	0.02
7	1.407	0.04	0.18	0.10	0.17	0.36	0.09	0.05	0.01
8	1.400	0.01	0.09	0.59	0.20	0.08	0.00	0.02	0.00

**Table 3:** Fitting parameters for the autocorrelation function.

pigment	1	2	3	4	5	6	7	8
*λ*	81.26	99.89	135.50	88.41	99.92	84.28	126.11	97.51
1/*γ*	11.41	18.94	15.31	13.32	16.88	16.95	14.52	21.19
1/*γ ′*	4.28	4.83	3.11	4.03	4.40	4.01	3.31	5.60

*λ* in units of cm^−1^, 1/*γ* and 1/*γ*′ in units of fs.
