# Supplementary material for: Hybrid QM/MM study of FMO complex with polarized protein-specific charge
Source: Sci Rep. 2015 Nov 27;5:17096. doi: 10.1038/srep17096 (PMC4661465; doi:10.1038/srep17096)
Supplement: Supplementary Information [file srep17096-s1.pdf]

Supplementary Information for "Hybrid QM/MM study of FMO complex with polarized protein-specific charge"

Xiangyu Jia[1], Ye Mei[1,2], John Z.H. Zhang[1,2], and Yan Mo[1,*]

[1]Center for Laser and Computational Biophysics, State Key Laboratory of Precision Spectroscopy and Department of Physics and Institute of Theoretical and Computational Science, East China Normal University, Shanghai 200062, China
[2]NYU-ECNU Center for Computational Chemistry at NYU Shanghai, Shanghai 200062, China
[*]ymo@phy.ecnu.edu.cn

Stable trajectory from MD simulations is critical to reliable spectrum calculations. Figure S1 shows the root mean square deviations of the FMO trimer in long time simulations utilizing AMBER charges (red) and PPC (black). The protein is more stable under PPC than it is under AMBER charges.

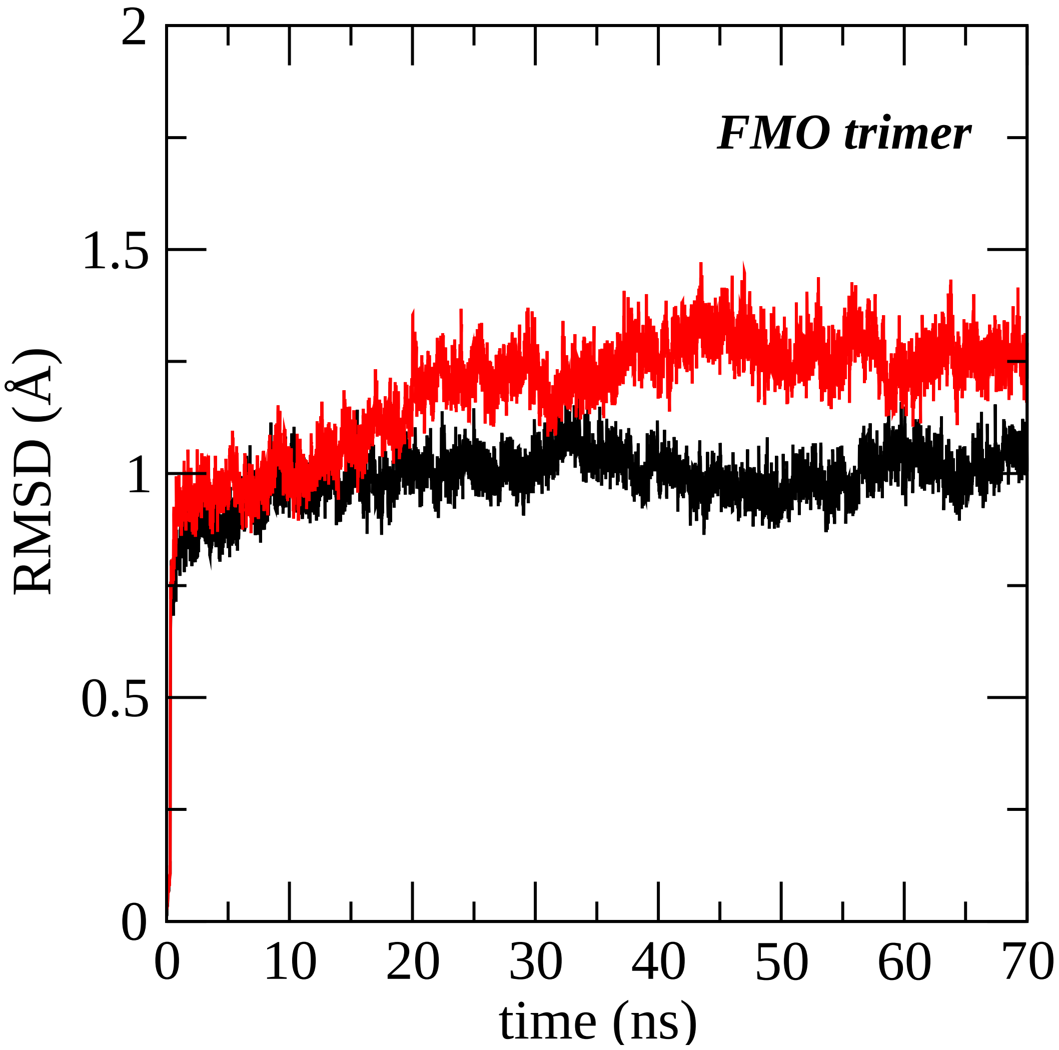

Figure S1. The variations of RMSD for FMO trimer during MD simulations based on PPC (black) and conventional mean-field AMBER charges (red).

Shown in Figure S2 are the site energies based on AMBER charges and PPC. The site energies show a wider distribution under PPC than that under AMBER charges. Especially, a 0.01 eV shift is seen for BChl 3.

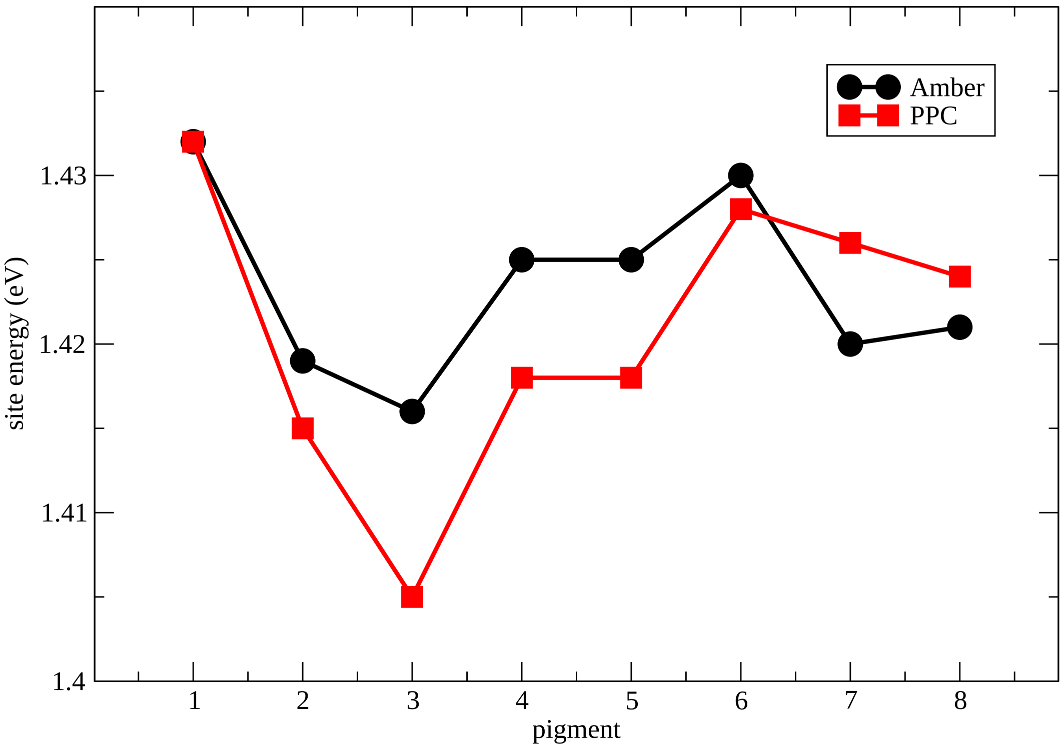

Figure S2. Site energies calculated by PPC and conventional mean-field charges.

The systematic error of ZINDO/S can be fixed by blue-shifting the site energies by 823 cm^{-1}. The absorption spectrum is then obtained by using the Lorentzian lineshape, as shown in Figure S3.

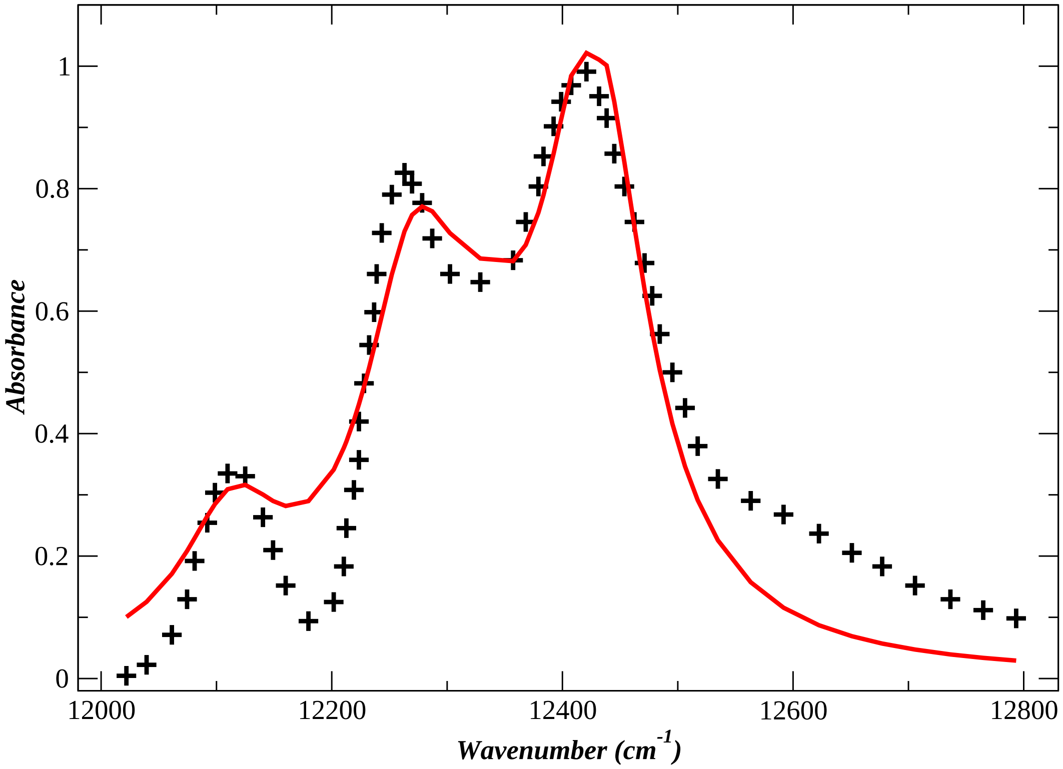

Figure S3. Simulated linear absorption spectrum (red line) compared with the experimental spectrum (black plus).

The site energies and their corresponding standard deviations over a 20-ps production run collected with a time interval of 0.5 fs and a 200-ps production run collected with a time interval of 5 fs are shown in Figure S4. As can be seen in this figure, both the site energies and their fluctuations from these two trajectories show high similarities.

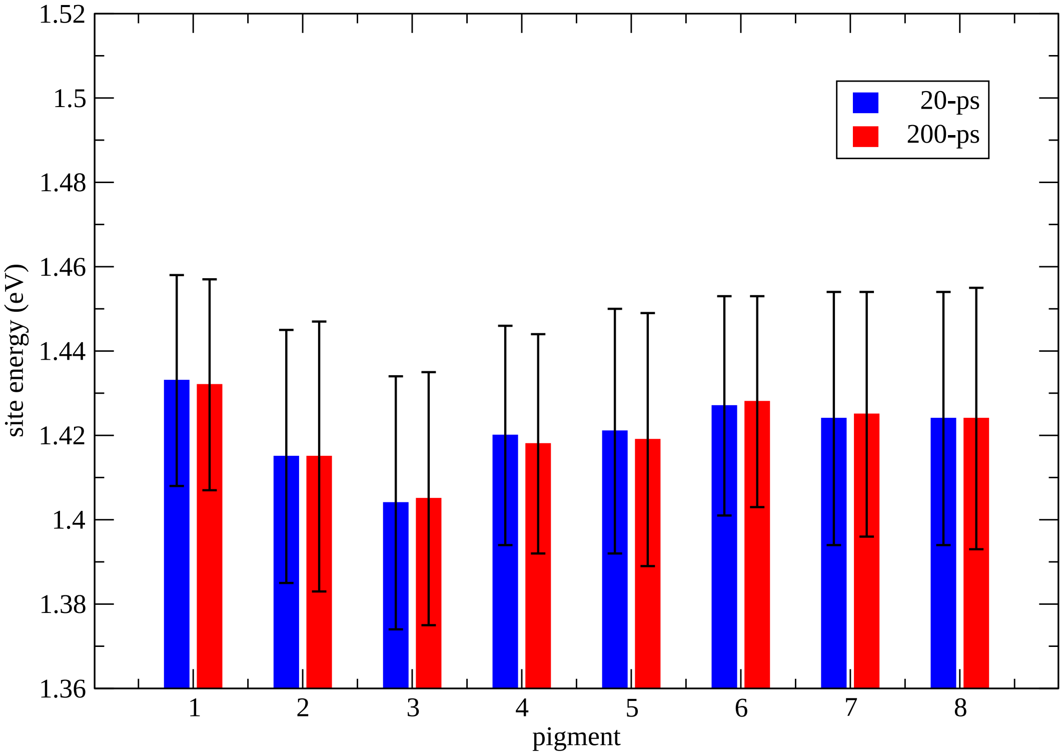

Figure S4. Site energies and their corresponding standard deviations over 20-ps production run (blue) and 200-ps production run (red).

In a previous study, Adolphs and Renger[1] estimated the spectral density based on the fluorescence line narrowing spectrum. Based on this spectral density, Nalbach et al.[2] added a single vibrational model. The spectral density with this additional model only leads to small differences in the exciton dynamics. The spectral density in our work is directly obtained by the QM/MM calculations based on a MD simulation. The comparison between our work and the above two mentioned spectral densities is shown in Figure S5.

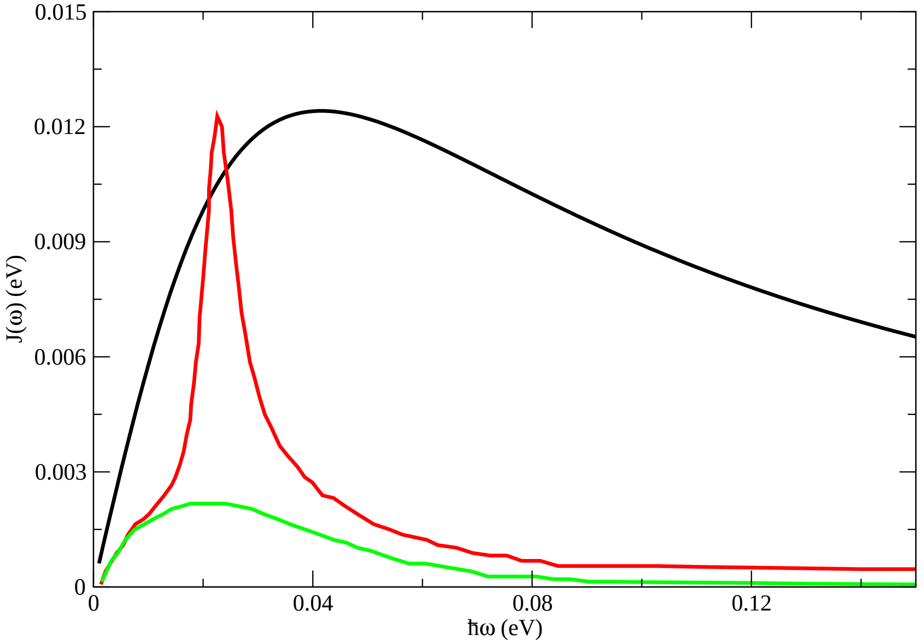

Figure S5. Spectral densities from our work (black), Adolphs et al.'s (green) and Nalbach et al.'s (red).

References

1. Adolphs, J. & Renger, T. How proteins trigger excitation energy transfer in the FMO complex of green sulfur bacteria. *Biophys. J.* **91**, 2778–2797 (2006).

2. Nalbach, P., Braun, D. & Thorwart, M. Exciton transfer dynamics and Quantumness of energy transfer in the Fenna-Matthews-Olson complex. *Phys. Rev. E* **84**, 041926 (2011).
